# Syringocystadenoma Papilliferum and Nevus Sebaceous of Jadassohn: Disentangling Rare Simultaneous Pathologies

**DOI:** 10.7759/cureus.89941

**Published:** 2025-08-12

**Authors:** Maria Troupi, Dimitra Daskalopoulou, Ameer Shehade, Theona Kiknadze, Chrysovalanti Oikonomidi, Dimosthenis Chrysikos, Theodore Troupis

**Affiliations:** 1 Department of Anatomy, National and Kapodistrian University of Athens School of Medicine, Athens, GRC; 2 Plastic and Reconstructive Surgery, Naval Hospital of Athens, Athens, GRC; 3 Dermatology, University of Athens, Athens, GRC

**Keywords:** adnexal tumors, differential diagnosis, nevus sebaceous of jadassohn, scalp tumor, syringocystadenoma papilliferum

## Abstract

Nevus sebaceous of Jadassohn (NSJ) is a cutaneous benign hamartoma that has been associated with the development of malignant cutaneous tumors such as basal cell carcinoma (BCC). Its coexistence with benign cutaneous tumors has also been reported, while differential diagnosis is considered challenging due to comparable characteristics. Syringocystadenoma papilliferum (SCAP) is a rare benign adnexal tumor and its coexistence with NSJ constitutes an unusual entity, which may lead to diagnostic uncertainty and warrants histopathological examination due to potential malignant transformation. We report a case of a syringocystadenoma papilliferum in a 42-year-old female which developed over a longstanding nevus sebaceous of Jadassohn, presenting as an ulcerated nodule encircled by a hairless plaque on the scalp, a clinical association that presents diagnostic challenges and can resemble malignant skin tumors. Herein, this case highlights the importance of clinical suspicion and complete surgical excision as well as histopathological evaluation of evolving lesions to establish diagnosis and prevent the development of malignant neoplasms.

## Introduction

Nevus sebaceous of Jadassohn (NSJ) is a benign hamartoma involving epidermis, pilosebaceous units, follicular and apocrine structures that is usually evident at birth or in early childhood [1,2]. At the initial stage of its development, it usually presents as a well-demarcated, yellowish, hairless plaque, which undergoes morphologic changes during adolescence due to hormonal influence, acquiring a verrucous structure. NSJs have been reportedly associated with the development of various benign cutaneous tumors and secondary neoplasms of both sebaceous and non-sebaceous lineages during adulthood [1-3].

Syringocystadenoma papilliferum (SCAP) constitutes an uncommon benign adnexal tumor of apocrine or eccrine sweat gland origin and appears at birth in 50% of cases. Differential diagnosis from other adnexal tumors is assumed challenging considering their overlapping clinical features and their common differentiation [4]. Herein, clinical suspicion is warranted, as definitive diagnosis and exclusion of associated malignancies can only be established through histopathological examination of the evolving tissues.

## Case presentation

**Figure 1:**
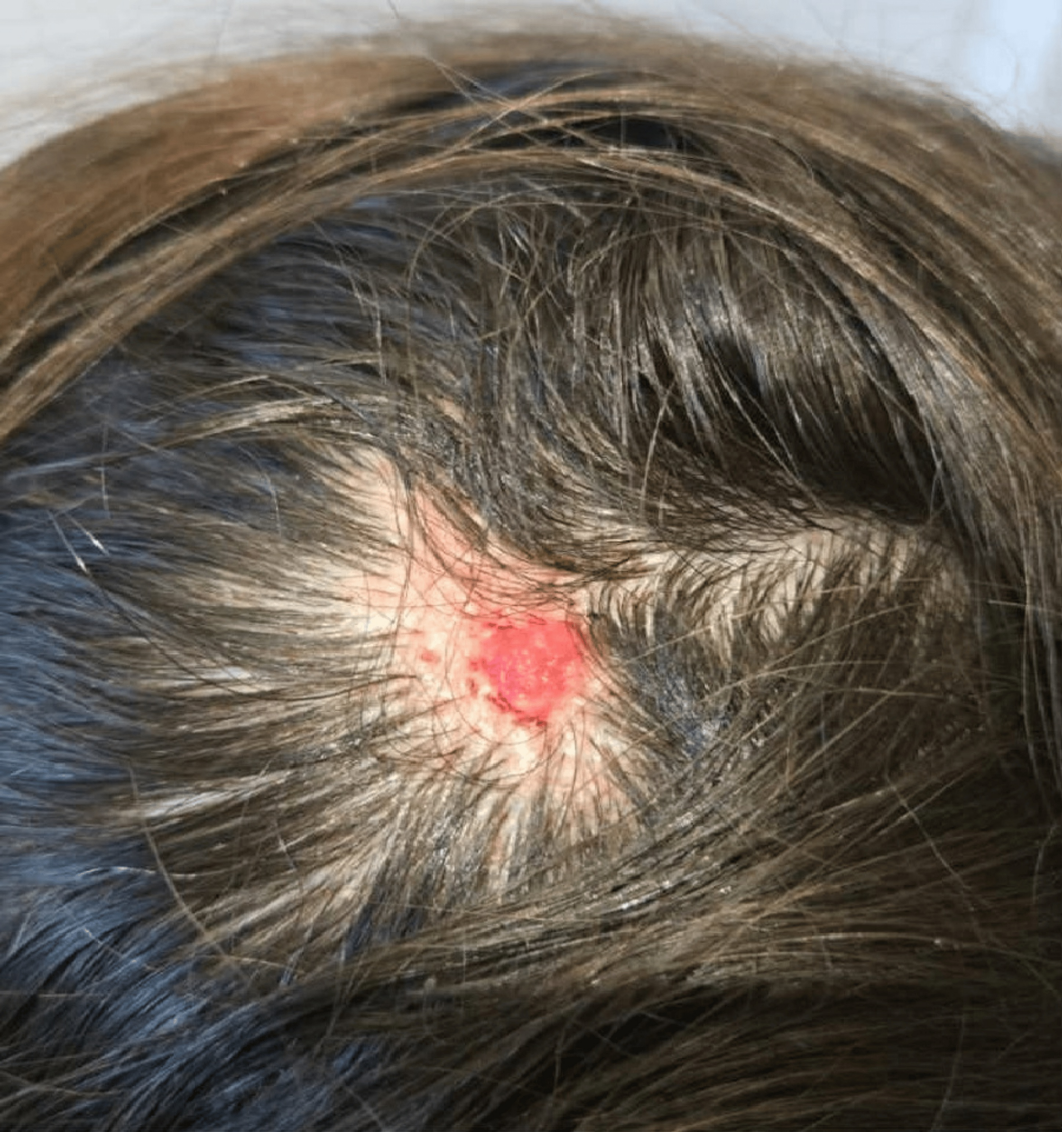
Preoperative image. Well-defined, hairless, yellowish plaque measuring 1.5 × 3.5 cm with verrucous changes encircling an exophytic, ulcerated pinkish nodule extending on the temporoparietal region of the scalp.

A 42-year-old female presented for evaluation of an alopecic plaque over her scalp since childhood as well as a slowly growing nodular formation which developed on the plaque over the last three years. The physical examination revealed a well-defined, hairless, yellowish plaque measuring 1.5 × 3.5 cm with verrucous changes and an exophytic, ulcerated, pinkish nodule extending on the temporoparietal region of the scalp (Figure 1). There was no regional lymphadenopathy or systemic symptoms. A clinical diagnosis of a basal cell carcinoma on the grounds of a nevus sebaceous was considered and the lesion was surgically removed with macroscopically negative resection margins.

**Figure 2:**
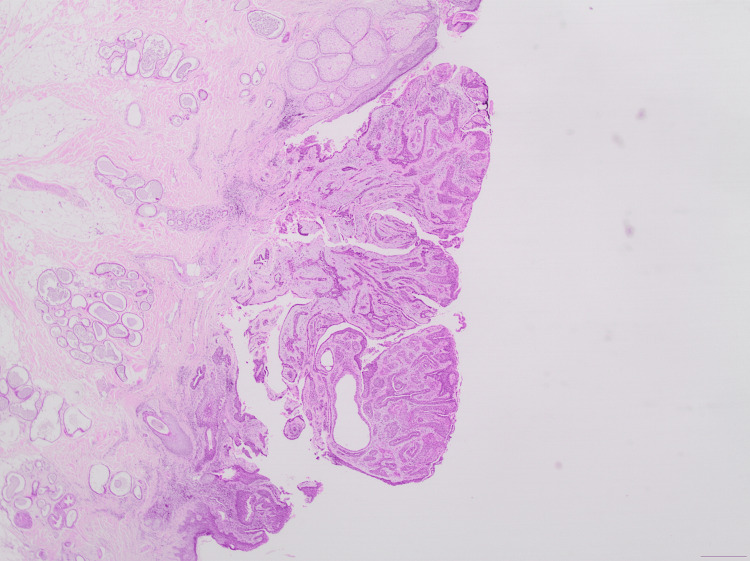
Glandular proliferation with prominent papillary architecture (magnification x20).

**Figure 3:**
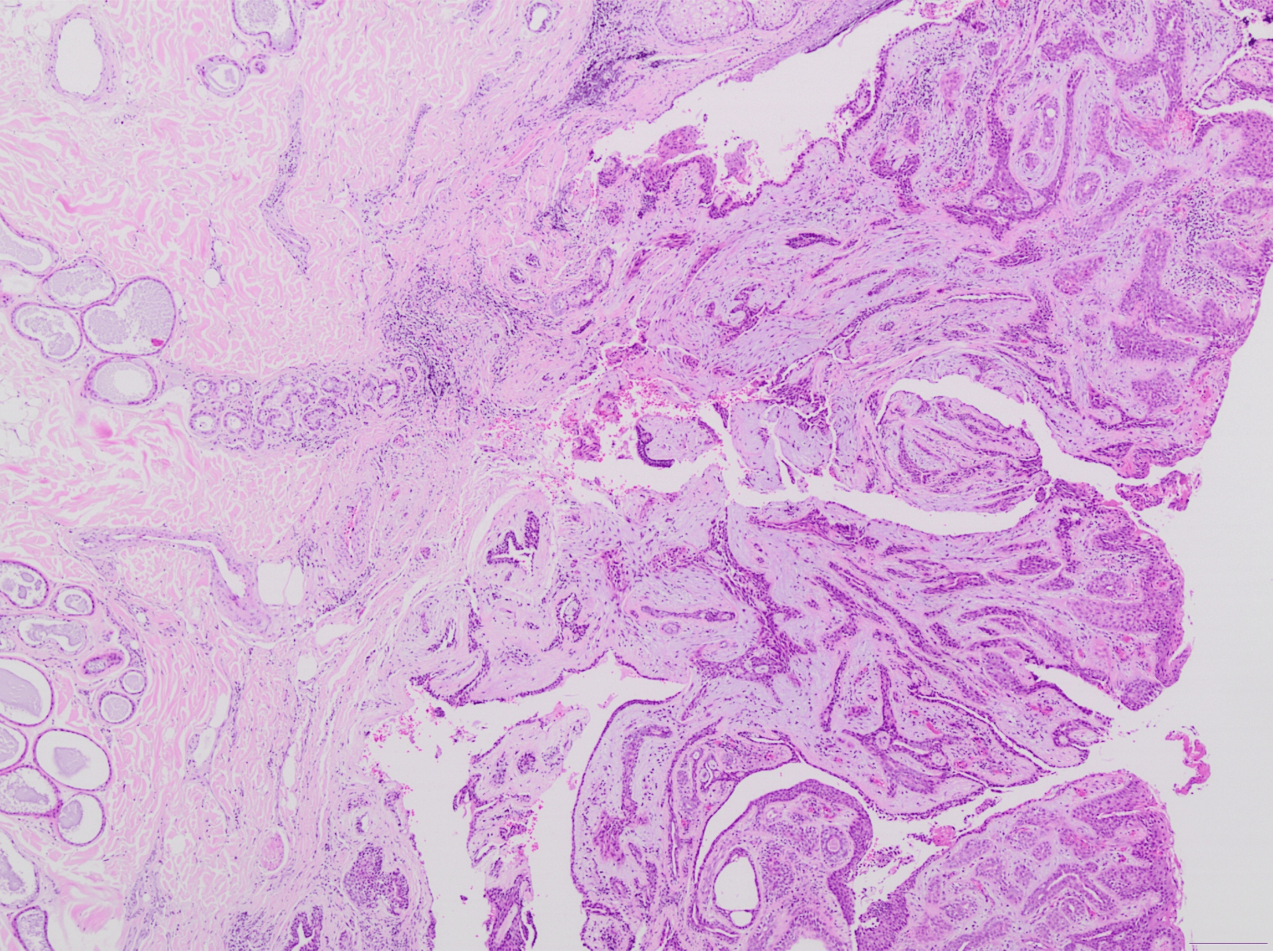
Exoendophytic proliferation of glands with cystic and papillary architecture and epidermal connection (magnification x40).

**Figure 4:**
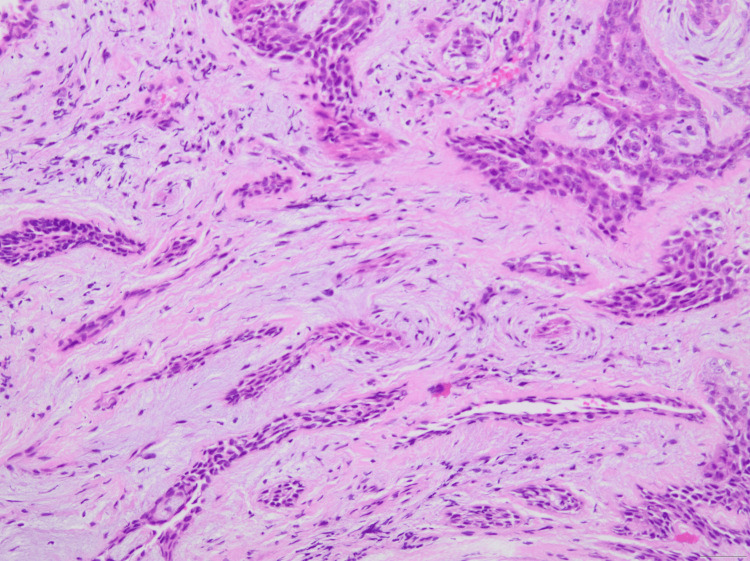
Glands with double layer of cuboidal and columnar cells in a background of myxoid and partly hyalinized stroma. Scattered plasma cells are identified (magnification x200).

**Figure 5:**
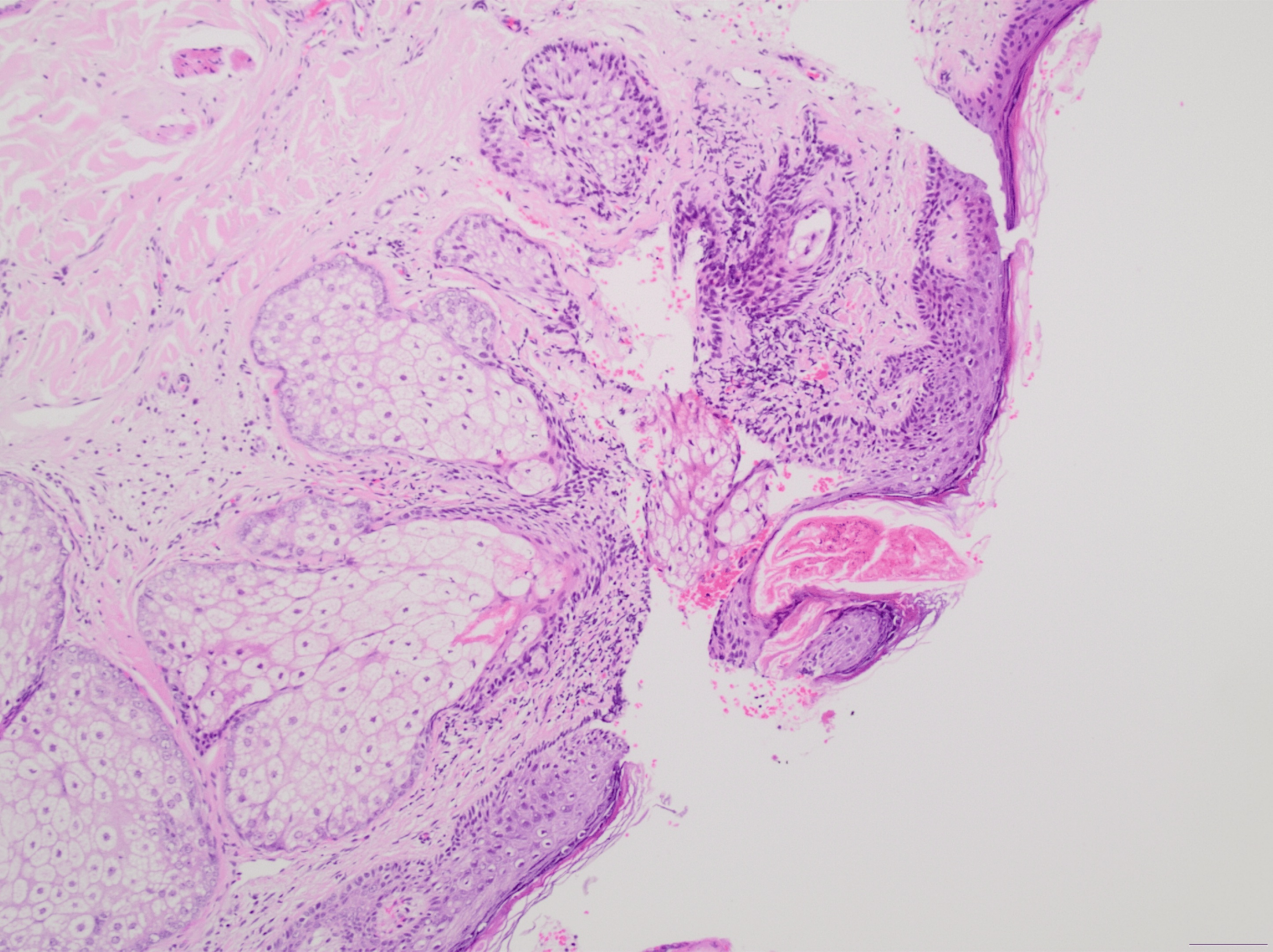
Lesion in association with an adjacent nevus sebaceous (magnification x100).

The histological examination revealed an exophytic lesion, characterized by cystic invaginations, which emerge from a papillomatous epidermis (Figures 2, 3). The cystic invaginations presented focal papilliform architecture and were lined with two rows of cuboidal or columnar cells with localized eosinophilic projections. Stroma was sparsely infiltrated by inflammatory cells including plasma cells (Figure 4). Hyperplastic sebaceous glands were identified adjacent to the main lesion as well as aggregates of cystically distended glands presenting apocrine morphological features (Figure 5). There was no evidence of malignancy. These findings were indicative of the formation of a syringocystadenoma papilliferum in the background of a preexisting sebaceous nevus of Jadassohn.

**Figure 6:**
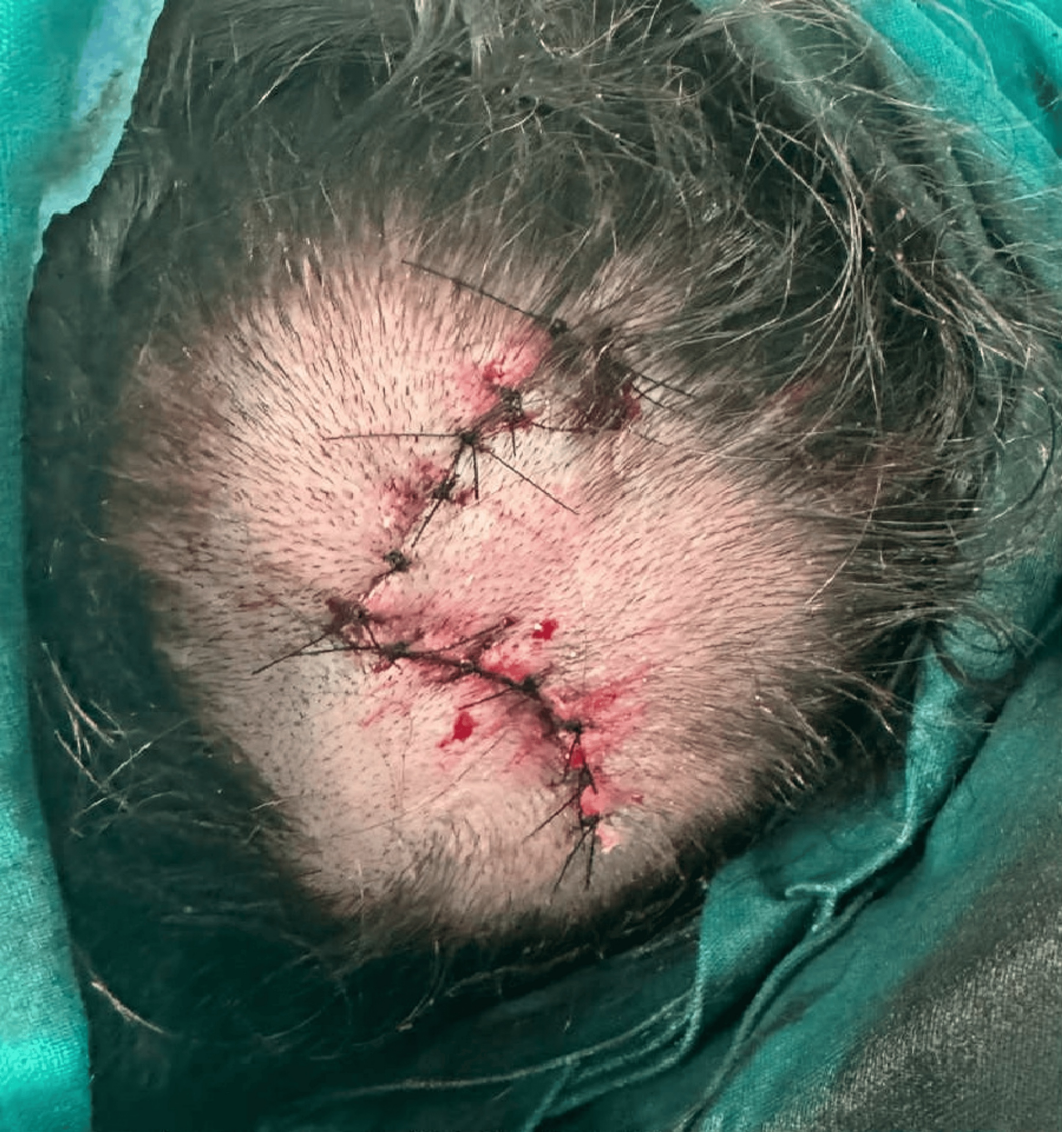
Postoperative image following complete excision of the lesion. The resulting defect was reconstructed using a local transposition “hatchet” type flap.

The lesions were excised in their entirety and the resulting defect was reconstructed using a local transposition “hatchet” type flap (Figure 6). The postoperative course of the patient was uneventful and no wound healing issues were encountered. The patient remains recurrence-free at the six-month follow-up examination.

## Discussion

Syringocystadenoma papilliferum is considered a childhood tumor and occurs mostly in the head and neck region and especially the scalp, although unusual locations with varied presentations such as forearm, arm, anterior chest wall and lateral abdominal wall have also been reported [5]. SCAP usually develops de novo or may arise within a preexisting nevus sebaceous. Its clinical significance emanates from its variability in clinical presentation and diagnostic complexity, as well as its potential to develop its malignant variant, syringocystadenocarcinoma papilliferum, or other cutaneous malignancies such as basal cell carcinoma and squamous cell carcinoma in 10% of the cases.

Three clinical presentations of SCAP have been encountered, including an alopecic plaque on the scalp, a linear variant incorporating papules or nodules extending to regions of the face and neck, and solitary nodules with a predisposition for development on the trunk [4]. Rapid growth or morphological changes mandate surgical excision and histopathological evaluation. Its definitive diagnosis is histopathologic, presenting with endophytic invaginations of the epidermis forming papillary projections lined by a dual-layered epithelium (luminal columnar and outer cuboidal/myoepithelial cells), along with stromal plasma cell infiltrates [6,7].

The origin of SCAP remains controversial, though it is considered to be a hamartomatous tumor arising from pluripotent cells. Immunohistochemically, the luminal cells express CK7, EMA, and CEA, consistent with apocrine differentiation; the basal/myoepithelial cells are positive for p63, SMA, and S100. Strong CD138 expression may highlight the plasma cell-rich stroma. According to an immunological and ultrastructural study by Yamamoto et al., SCAP exhibits microscopic features of both eccrine and apocrine glands, consisting of several cell types corresponding to various developmental stages from primitive clear cells, suggesting stem or progenitor cell properties to basal cells [8].

NSJ constitutes a benign hamartoma that has been associated with the development of secondary benign and malignant tumors from different lineages of differentiation. According to a retrospective study by Idriss et al., secondary neoplasms were ascertained in 21.4% of the cases, while malignancy was reported in 2.5% of the patients [9,10]. Trichoblastoma was the most common benign tumor that coexisted with NSJ, while the development of SCAP had also been reported. In the context of secondary malignancy, basal cell carcinoma has been reported in most of the studies, while sebaceous carcinoma has also been described in the background of NSJ plaques [10].

Kuo et al. presented a rare case of seven secondary tumors, namely syringocystadenoma papilliferum, desmoplastic trichilemmoma, sebaceoma, trichoblastoma, pigmented trichoblastoma, sebaceous adenoma, and tumor of follicular infundibulum arising in the background of NSJ [2]. The coexistence of multiple tumors arising from an NSJ is considered the outcome of proliferation of pluripotential primary epithelial germ cells and development of variable cell entities [11]. Histopathological analysis of multiple sections of the lesion remains the milestone in the differential diagnosis and appropriate treatment of these tumors, which may emanate from different lineages, further supporting the theory that NSJ evolves from pluripotent primary epithelial germ cells that may also give rise to a variety of tumors.

The clinical presentation of SCAP mimics the corresponding characteristics of basal cell carcinoma (BCC) and has been speculated to constitute an intermediate step from NSJ to BCC [12]. Kneitz et al. also presented the histopathological evolution of invasive syringocystadenocarcinoma papilliferum that developed from NSJ through transitional stages including SCAP and syringocystadenocarcinoma papilliferum in situ [13].

Our case report further highlights the necessity of surgical excision and complete histopathological evaluation of such lesions, as clinical diagnosis may be misleading considering their overlapping characteristics. Early excision of these lesions may also be warranted establishing the appropriate management of potential malignancies or even avoiding the sequential development of invasive counterparts.

## Conclusions

Syringocystadenoma papilliferum arising in nevus sebaceous of Jadassohn is a rare phenomenon and clinicians should maintain a high index of suspicion for neoplastic transformation, especially with secondary changes during puberty or adulthood. This case highlights the diagnostic challenges that accompany the coexistence of such lesions which can resemble malignant skin tumors as well as the importance of histopathological evaluation in the establishment of diagnosis. Complete surgical excision and further management according to the histopathological findings remain the cornerstones of management to avoid unwanted sequelae such as an impending neoplastic transformation.
